# Metabolic miracle or misguided shift? Tirzepatide in the era of obstructive sleep apnea (OSA)

**DOI:** 10.1097/MS9.0000000000004135

**Published:** 2025-10-28

**Authors:** Devya Khaim Chandani, Harshika Khaim Chandani, Muhammad Saad Khan, Syeda Fadak Zahra Hujjat, Aminath Waafira

**Affiliations:** a Department of Medicine, Shaheed Mohtarma Benazir Bhutto Medical College (SMBBMC), Karachi, Pakistan; b Department of Medicine, Jinnah Sindh Medical University, Karachi, Pakistan; c Department of Medicine, Allama Iqbal Medical College, Lahore, Pakistan; d School of Medicine, The Maldives National University, Malé, Maldives

**Keywords:** CPAP, GLP-1 receptor agonist, obstructive sleep apnoea, tirzepatide, weight loss therapy

## Abstract

Obstructive sleep apnoea (OSA) is a prevalent disorder marked by recurrent upper airway obstruction during sleep, often linked to obesity and poor continuous positive airway pressure (CPAP) adherence. Tirzepatide, a dual GIP and GLP-1 receptor agonist approved for weight loss, has garnered interest as a potential pharmacologic option for OSA due to its weight-reducing effects. While early studies show modest reductions in apnoea-hypopnoea index and improved sleep quality, these benefits are indirect and fail to address the anatomical basis of OSA. Moreover, concerns about long-term efficacy, weight regain post-discontinuation, and adverse effects such as gastrointestinal symptoms and pancreatitis limit its standalone use. Tirzepatide may complement, but not replace, first-line therapies like CPAP. Future research should assess its role within integrated treatment models and clarify its impact on airway mechanics. Caution is warranted to avoid overreliance on pharmacologic solutions at the expense of established, effective interventions in managing this multifactorial condition. Further research should evaluate the integrated models that combine pharmaceutical therapy with lifestyle and mechanical therapies, investigate the direct impact on airway mechanics, and use the artificial intelligence to identify responders and optimize personalized treatment strategies.

*To the Editor,*

Obstructive sleep apnea (OSA) is a chronic disorder marked by repeated upper airway obstruction during sleep, leading to intermittent hypoxia, disrupted rest, and heightened chances of cardiometabolic impacts. Several clinical signs, such as extreme tiredness during the day, reduced neurocognitive ability, mood changes, and diminished quality of life, are linked with it^[1]^. The increasing rates globally are mainly due to obesity, aging population and sedentary lifestyles. Recent worldwide epidemiological data reveal that out of 936 million individuals with mild to moderate OSA, 425 million people between the ages of 30 and 69 have severe forms requiring intervention, making this condition an important public health issue. It substantially increases the risk of type 2 diabetes, cardiovascular disease, stroke, metabolic syndrome, and all-cause mortality. Beyond its health impact, untreated OSA contributes to higher healthcare costs, reduced productivity, and increased accident risk, underscoring the urgent need for more effective and sustainable management strategies^[2]^.

**HIGHLIGHTS**

- Tirzepatide, a dual GIP/GLP-1 receptor agonist, has shown modest benefits in reducing OSA severity through weight loss.
- These effects are indirect and do not address the anatomical causes of OSA, limiting its standalone utility.
- Concerns remain regarding long-term efficacy, weight regain post-discontinuation, and potential adverse effects such as pancreatitis and thyroid complications.
- Tirzepatide should be viewed as a complementary option, especially for patients with obesity-related OSA who are non-adherent to CPAP.

## Current treatment landscape and limitations

A variety of therapies are used to treat OSA, and each has advantages and disadvantages of its own. Regardless of body weight, continuous positive airway pressure (CPAP) is the gold standard for quickly and reliably relieving airway blockage. However, discomfort, inconvenience, and mask intolerance are major obstacles that prevent long-term compliance. In mild-to-moderate OSA, oral appliances, such as mandibular advancement splints, provide a less invasive option and have been shown to be effective; however, in severe cases, their effectiveness decreases, and they may cause temporomandibular discomfort. Many patients, particularly those with structural deformities, may benefit from upper airway surgery; however, results are not always consistent, and the risks are significant^[3]^. Although patient compliance is unpredictable, lifestyle modification, which includes weight loss, exercise, and alcohol abstinence, is crucial and maintaining long-term weight loss is notoriously challenging.

All of these disadvantages point to the unmet demand for supportive and supplemental therapies. Pharmacologic strategies aimed at obesity-related OSA have attracted attention as viable substitutes for current treatments. This manuscript has been developed in accordance with the TITAN guidelines (Table 1)^[4]^.

**Table 1:** TITAN guideline checklist 2025

Topic	Item	Description	Page number
**Artificial Intelligence (AI) (some journals may prefer this in the methods and/or acknowledgments section and it should also be declared in the cover letter)**	5	**Declaration of whether any AI was used in the research and manuscript development**	No
		**If no, proceed to item 6.**	
		**If yes, proceed to item 5a**	
	5a	**Purpose and scope of AI use**	
		Precisely state why AI was employed (e.g., development of research questions, language drafting, statistical analysis/summarization, image annotation, etc.). Was generative AI utilized and if so, how? Clarify the stage(s) of the reporting workflow affected (planning, writing, revisions, figure creation). Confirmation that the author(s) take responsibility for the integrity of the content affected/generated.	
	5b	**AI tool(s) and configuration**	
		Name each system (vendor, model, major version/date). State the date it was used Specify relevant parameters (e.g., prompt length, plug-ins, fine-tuning, temperature). Declare whether the tool operated locally on-premises, or via a cloud API and any integrations with other systems.	
	5c	**Data inputs and safeguards**	
		Describe categories of data provided to the AI (patient text, de-identified images, literature abstracts). Confirm that all inputs were de-identified and compliant with GDPR/HIPAA. Note any institutional approvals or data-sharing agreements obtained.	
	5d	**Human oversight and verification**	
		Identify the supervising author(s) who reviewed every AI output. Detail the process for fact-checking, clinical accuracy checks State whether any AI-generated text/figures were edited or discarded. Acknowledge the limitations of AI and its use	
	5e	**Bias, ethics, and regulatory compliance**	
		Outline steps taken to detect and mitigate algorithmic bias (e.g., cross-checking against under-represented populations).	

## Recent FDA approval of tirzepatide: mechanism and clinical evidence

Tirzepatide (zepbound), a dual glucose-dependent insulinotropic polypeptide (GIP) and glucagon-like peptide-1 receptor agonist (GLP-1), was initially developed for type 2 diabetes and later approved for weight management. Increasing satiety, decreasing caloric intake, and improving insulin sensitivity result in significant weight loss^[5]^. With a half-life of approximately 5 days, it is typically administered subcutaneously, enabling once-weekly dosing to maintain steady therapeutic levels^[6]^. Obesity has been suggested as a possible treatment option because it is the most significant modifiable risk factor for OSA. Given this, the recent FDA approval of tirzepatide has sparked a lot of excitement and media coverage as a potentially “game-changing” pharmaceutical option; however, before such enthusiasm is warranted, its role in managing OSA necessitates a careful evaluation of the supporting data.

Initial trials suggest small decreases in the apnoea-hypopnoea index (AHI) and enhanced sleep architecture in people who lose significant weight with the drug^[7]^. However, these effects are secondary and not related to the upper airway pathology that causes OSA. This indirect way of action poses a significant therapeutic constraint. OSA is primarily a mechanical airway condition rather than a metabolic problem; however, the two can overlap^[7]^. While losing weight can lower the severity of OSA, it seldom cures the condition, particularly in people with anatomical or neuromuscular predispositions^[8]^. Using a metabolic medication to treat a structural issue results in a mismatch between mechanism and disease target.

## Safety, sustainability, and clinical constraints

The absence of long-term data on AHI following tirzepatide cessation is a critical limitation, as weight regain is a common occurrence after discontinuing weight loss medications, potentially leading to a recurrence of OSA symptoms. A systematic review and meta-analysis revealed that individuals who discontinued semaglutide or tirzepatide experienced an average weight regain of 9.69 kg^[9]^. This rapid weight regain may negate the metabolic benefits initially observed during treatment^[10]^.

Moreover, the safety profile of tirzepatide cannot be overlooked. Side effects include nausea, vomiting, and diarrhea; acute pancreatitis, cholelithiasis, or cholecystitis, and possible thyroid tumor growth add further complexity to long-term usage^[11,12]^. Patients with OSA are often multimorbid, and adding another chronic medication with potential systemic risks requires careful clinical judgement.

## Adjunctive role and integrated management models

Tirzepatide may be best used as an adjunct in the context of comprehensive OSA care rather than as a substitute. Pharmacological weight loss will lessen the degree of disease in patients who are not responsive to CPAP and can augment results with mandibular devices or positional therapy. Tirzepatide may improve general outcomes and cardiometabolic comorbidities among obese users of CPAP, adding to rather than substituting the gold-standard treatment. Long-term weight gain may be reduced by tirzepatide, which will assist in producing longer results when combined with structured behavioral programs. The optimal way forward is anticipated to be integrated management strategies combining medication, mechanical therapies, and lifestyle interventions^[13]^.

## Artificial intelligence: a paradigm shift in OSA and pharmacotherapy research

Given its revolutionary potential, artificial intelligence (AI) has a bright future in clarifying tirzepatide’s role in OSA. Conventional parameters, such as weight loss or AHI decline, only provide a limited view of the therapeutic effect. AI-driven approaches that use machine learning to integrate multi-omics data, clinical phenotypes, and upper airway imaging can help predict adherence, find new response indicators, and identify patients who are most likely to benefit. Multi-omics analysis could classify responders based on metabolic profiles, while AI-driven imaging could evaluate structural airway changes after pharmacologically induced weight loss.

AI models like AlphaFold have revolutionized structural biology and drug discovery, going beyond patient stratification^[14,15]^. Large language models are also performing well in clinical decision support and medical diagnostics. By directing integrated therapy design and dynamic treatment pathways, integrating these tools into OSA research may hasten the transition away from empirical, one-size-fits-all approaches and toward precision medicine.

## Future directions

Well-designed trials investigating the appropriateness of tirzepatide when added to proven therapies such as CPAP, mandibular advancement devices or upper airway stimulation are needed. There is a need for longitudinal studies to determine the duration of OSA improvement after discontinuation of medication and to develop strategies that prevent rebound weight gain. In addition to weight loss, studies need to investigate in a mechanistic manner whether tirzepatide has a direct effect on upper airway neuromuscular tone. Finally, AI-guided approaches could enhance precision therapy in the field by pinpointing optimal patient subgroups, predicting therapy response and designing personalized models of OSA management.

## Conclusion

In conclusion, tirzepatide represents a welcome pharmacologic advance in the management of OSA that is related to obesity; however, its effects appear to be largely weight-related and are not intended to replace efficacious mechanical therapies. Prudent applications are necessary due to safety, compliance and therapeutic oversimplifications. An integrated, multimodal approach that involves behavioral, mechanical and pharmacologic interventions with AI-enabled precision medicine will yield long-lasting, personalized results for treating OSA.

## Data Availability

No data were generated for this manuscript.

## Research registration unique identifying number (UIN)

(1) Name of the registry: N/A. (2) Unique Identifying number or registration ID: N/A. (3) Hyperlink to your specific registration (must be publicly accessible and will be checked): N/A.
